# Ultrasound-derived tibia-fascia angle for noninvasive assessment of intracompartmental pressure in tibial plateau fractures

**DOI:** 10.1371/journal.pone.0344990

**Published:** 2026-03-25

**Authors:** Heng Zhang, Luqi Li, Huiyang Jia, Haofei Wang, Qi Dong, Jialiang Guo, Heping Deng, Zhiyong Hou

**Affiliations:** 1 Department of Orthopaedic Surgery, Hebei Medical University Third Hospital, Shijiazhuang, Hebei, China; 2 Engineering Research Center of Orthopedic Minimally Invasive Intelligent Equipment, Ministry of Education, Shijiazhuang, Hebei, China; 3 Department of Ultrasound Medicine, Hebei Medical University Third Hospital, Shijiazhuang, Hebei, China; Southern Medical University Nanfang Hospital, CHINA

## Abstract

**Background:**

Current noninvasive diagnostics for acute compartment syndrome (ACS) lack clinical practicality and reliability. This study aimed to evaluate ultrasound-derived tibia-fascia angle (TFA) as a novel morphometric surrogate for intracompartmental pressure (ICP) assessment.

**Methods:**

In this observational study, 105 patients with closed tibial plateau fractures were enrolled at a tertiary trauma center. TFA was bilaterally measured using B-mode ultrasound by blinded operators. Invasive ICP served as the reference standard. Correlations were analyzed via Spearman correlation analysis and multivariable linear regression, and diagnostic performance for high compartment pressure (HCP, ICP > 30 mmHg) was assessed using ROC analysis.

**Results:**

ΔTFA showed a significant linear correlation with ICP after adjustment for confounders (*β* = 1.74 mmHg/°, 95% CI: 1.07–2.41, *P*adj < 0.001) and a moderate monotonic association (*ρ* = 0.545, 95%CI: 0.395–0.667, *P <* 0.001). For detecting HCP, ΔTFA achieved an area under the receiver operating characteristic curve (AUC) of 0.716 (95% CI: 0.621–0.812), with 86.5% sensitivity and 52.9% specificity at the optimal cutoff (ΔTFA ≥ 4.9°).

**Conclusion:**

ΔTFA provides a rapid, operator-friendly method for noninvasive ICP estimation, demonstrating moderate diagnostic accuracy as a screening tool for HCP in tibial plateau fractures. Its integration into trauma workflows could enhance early risk stratification, particularly for high-energy fractures, though specificity limitations warrant complementary confirmatory tests.

## Introduction

Acute compartment syndrome (ACS) constitutes a surgical emergency that can arise from various etiologies, including fractures, crush injuries, and burns [1]. High-energy trauma triggers soft tissue edema and rapid increase in intracompartmental pressure (ICP), compromising capillary blood flow and tissue oxygenation while promoting metabolite accumulation, ultimately leading to irreversible muscle and nerve damage [2–4]. Ischemia lasting 3 hours may result in muscle necrosis, 4 hours in neuropraxia, and 8 hours in axonotmesis [5]. Importantly, although elevated or high compartment pressure does not in itself constitute a clinical diagnosis, it represents a key pathophysiological driver underlying the development of ACS. The lower leg is the most common site for ACS due to its rigid osseofascial confinement [6]. Emergency fasciotomy remains the sole definitive treatment [7,8], however, diagnostic delays significantly increase the risks of infection, permanent functional deficit, amputation, and mortality [4,8–11].

Despite its urgency, the clinical diagnosis of ACS remains challenging. Traditional physical examination findings, often summarized by the “5 Ps” (pain, paresthesia, pallor, pulselessness, paralysis), are frequently unreliable. Pallor and pulselessness, for instance, are indicative of arterial injury rather than compartmental hypertension and typically present only in late-stage ACS. While pain disproportionate to the injury and pain on passive stretch are more sensitive early indicators, their assessment is inherently subjective and often unobtainable in obtunded or polytraumatized patients [12–14]. Consequently, invasive ICP monitoring is often utilized as an objective adjunct. However, this modality is limited by its invasive nature, potential for iatrogenic infection, and technical difficulties in continuous monitoring at fracture sites [12,15–17].

Given these limitations, noninvasive diagnostic alternatives, including ultrasound [14,17], near-infrared spectroscopy [18,19], magnetic resonance imaging [20,21], quantitative tissue hardness measurement [22], and bioimpedance [23], have been explored.. Among these, ultrasound offers distinct advantages regarding accessibility, cost-effectiveness, and the capability for real-time soft tissue imaging [1,24]. Recently, Mühlbacher et al. [25] proposed a novel morphometric parameter, the tibia-fascia angle (TFA), based on the hypothesis that increased ICP would displace the fascia away from the anterolateral tibial cortex. Their cadaveric study demonstrated a strong correlation between the TFA and ICP, with each 1° increase in TFA corresponding to a 3.9 mmHg ICP rise (95% CI: 3.8–4.0; *P* < 0.001). However, the clinical validity of this ultrasound-based metric in living subjects remains unverified.

Building on these foundations, this study aims to evaluate the correlation between ultrasound-derived TFA and ICP in patients with tibial plateau fractures, assessing the utility of TFA as a rapid, noninvasive tool for the early risk stratification and diagnosis of ACS.

## Methods

### Study design and participants

This observational study was conducted at a tertiary trauma center in China between October 2024 and June 2025. We consecutively enrolled patients aged 18–65 years who admitted with acute closed tibial plateau fractures within 48 hours of injury. The exclusion criteria were: (1) polytrauma or concomitant fractures at other anatomical sites; (2) pre-existing neuromuscular pathologies; (3) compromised soft tissue integrity at the measurement site; (4) inability to tolerate the required positioning for assessment; and (5) declined participation.

Based on preliminary data from Mühlbacher et al. [25], an a priori power analysis (power = 0.8; α = 0.05) indicated a minimum sample size of 89 participants. The study protocol adhered to the Declaration of Helsinki and was approved by the Institutional Review Board of the Third Hospital of Hebei Medical University (Approval No. Ke2023-107-2). Written informed consent was obtained from all participants prior to inclusion.

### Clinical information

Demographic and clinical data were extracted from electronic medical records. Demographic characteristics included sex, age, and body mass index (BMI, kg/m²). Fracture type was classified according to Schatzker classification, and further stratified into simple (Schatzker Ⅰ-Ⅲ) and complex (Schatzker Ⅳ-Ⅵ) fractures [26]. Injury mechanisms were categorized into four types: falls, blunt trauma, bicycle or electric bicycle accident, and motor vehicle accident [27].

### Ultrasound examination

All ultrasound examinations were performed by a qualified sonographer with over 3 years of experience, who was blinded to the patients’ clinical status. Imaging was conducted using an Aixplorer ultrasound system (Supersonic Imagine, Aix-en-Provence, France) equipped with an SL10–2 linear array transducer (6–10 MHz). Patients were positioned supine with the lower limb in neutral extension and muscles fully relaxed. The imaging site was standardized to the proximal 30% of the distance between the fibular head and the lateral malleolus. A generous amount of coupling gel was applied to prevent transducer-induced compression of the fascia.

**Fig 1:**
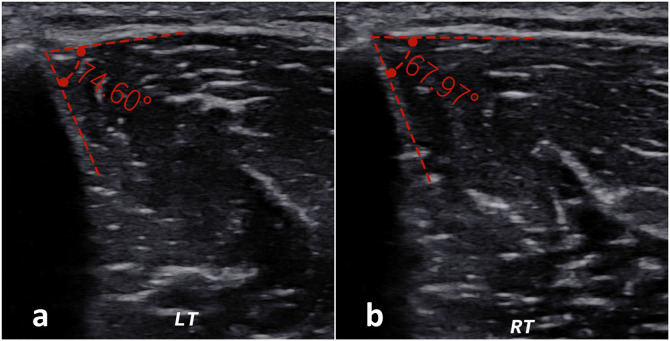
Ultrasound measurement of the tibia-fascia angle (TFA). Representative images are from a 40-year-old male with a Schatzker type II tibial plateau fracture sustained in an electric bicycle accident (intracompartmental pressure: 28 mmHg). (a) Affected side (left) showing a TFA of 74.60°; (b) Contralateral (unaffected) side showing a TFA of 67.97°. The TFA is defined as the angle subtended by the anterolateral tibial cortex and the tangent to the anterior compartment fascia, with the vertex at the fascial attachment.

Bilateral examinations were performed sequentially under consistent imaging parameters. Transverse B-mode scans of the anterior compartment were obtained with the transducer oriented perpendicular to the anteromedial tibial border. The TFA was defined as the angle subtended by the anterolateral tibial cortex and the tangent of the deep fascia, with the vertex located at their intersection (Fig 1). Images were digitally archived for offline analysis. Image quality was independently validated by a senior sonographer. Subsequently, two trained sonographers, blinded to both clinical data and ICP values, independently analyzed the archived images to measure TFA. The tibia-fascia angle difference (ΔTFA) was calculated as the TFA of the affected limb minus that of the contralateral unaffected limb.

### Intracompartmental pressure measurement

ICP was quantified at the precise anatomical location of the ultrasound assessment by an independent physician blinded to sonographic findings. Immediately following the ultrasound examination, ICP measurements were obtained using Stryker Intra-Compartmental Pressure Monitor (STIC; Stryker, Kalamazoo, MI) [28–30]. Following system assembly and zero-calibration, the needle was introduced into the anterior compartment under sterile conditions. Hydraulic continuity was established via injection of 0.3 ml normal saline, and the equilibrium pressure was recorded upon stabilization. According to ICP, patients were divided into high compartment pressure (HCP, ICP > 30 mmHg) group or non-high compartment pressure (Non-HCP, ICP ≤ 30 mmHg) group [13,31].

### Statistical analysis

Continuous and categorical variables were reported as mean ± SD/median (IQR) and frequencies, respectively. Group comparisons utilized Student’s t, Mann-Whitney U, or Chi-square/Fisher’s exact tests. TFA reliability was assessed via intraclass correlation coefficients (ICC), with ICC > 0.75 indicating excellent agreement [32]. The ΔTFA–ICP relationship was evaluated using Spearman’s rank correlation (*ρ*), with correlation strengths categorized as weak (<0.4), moderate (0.4–0.7), or strong (>0.7) [33]. Multiple linear regression analysis was performed to examine the linear relationship between ΔTFA and ICP, adjusting for age, sex, BMI, Schatzker classification, and injury mechanism. Interaction terms were included to assess effect modification, with robust methods applied where assumptions were violated. Subgroup analyses stratified by sex and Schatzker classification were performed using adjusted linear models, with Bonferroni correction for multiple comparisons. Diagnostic performance of ΔTFA for identifying HCP was evaluated using receiver operating characteristic (ROC) curve analysis and Youden’s index. Analyses were conducted using R (v4.2.0) and SPSS (v29.0.1), with significance defined as two-tailed *P* < 0.05.

## Results

### Participant characteristics and measurement reliability

**Fig 2:**
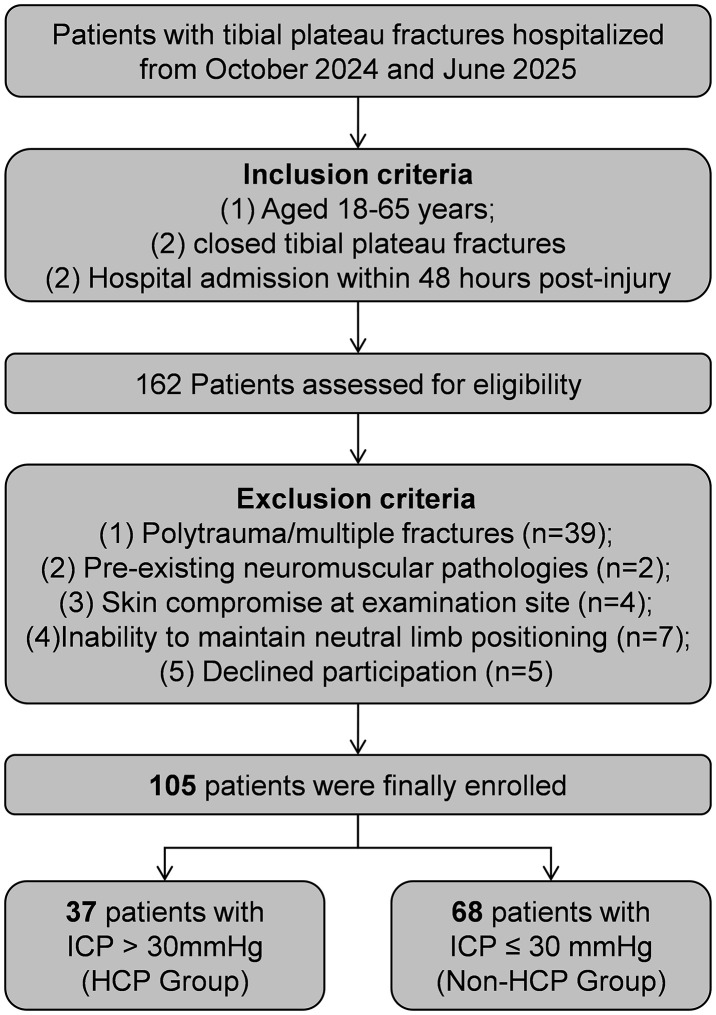
Flow diagram of included patients.

The study enrolled 105 patients with tibial plateau fractures (63.8% male, median age 45 years; Fig 2). Participants were stratified by ICP into Non-HCP (n = 68) and HCP (n = 37) groups. The HCP cohort was significantly younger (median: 43 vs 48 years, *P* = 0.026) and exhibited markedly higher ΔTFA values compared to the Non-HCP group (7.5° vs 4.7°, *P* < 0.001). A trend toward a higher prevalence of complex fractures (Schatzker Ⅳ-Ⅵ) was observed in the HCP group (73.0% vs 51.5%,), though this did not reach statistical significance (*P* = 0.053). Sex, BMI, contralateral TFA, and injury mechanism were comparable between groups (all *P* > 0.05; Table 1).

**Table 1:** Baseline characteristics of the study participants.

Variable	Total (n = 105)	Non-HCP group (n = 68)	HCP group (n = 37)	*P*-value*
**Sex, n (%)**				>0.999
Female	38 (36.2)	25 (36.8)	13 (35.1)	
Male	67 (63.8)	43 (63.2)	24 (64.9)	
**Age (years), median (IQR)**	45 (38, 53)	48 (40, 57)	43 (35, 50)	**0.026**
**BMI (kg/m²), mean ± SD**	25.9 ± 4.0	26.12 ± 4.0	25.49 ± 4.1	0.454
**Injury mechanisms, n (%)**				0.089
Fall	34 (32.4)	24 (35.3)	10 (27.0)	
Blunt trauma	9 (8.6)	3 (4.4)	6 (16.2)	
Electric bicycle accident	30 (28.6)	17 (25.0)	13 (35.1)	
Motor vehicle accident	32 (30.5)	24 (35.3)	8 (21.6)	
**Schatzker classification, n (%)**				0.053
Ⅰ-Ⅲ	43 (41)	33 (48.5)	10 (27.0)	
Ⅳ-Ⅵ	62 (59)	35 (51.5)	27 (73.0)	
**Affected side TFA (°),** **mean ± SD**	69.9 ± 5.2	69.3 ± 5.5	71.2 ± 4.6	0.053
**Contralateral side TFA (°), mean ± SD**	63.3 ± 4.6	63.6 ± 4.7	62.9 ± 4.6	0.496
**ΔTFA (°), median (IQR)**	5.9 (3.9, 8.2)	4.7 (3.6, 7.3)	7.5 (5.3, 10.3)	**<0.001**

*P-value<0.05 was considered for statistical significance (significant values in bold). IQR, Interquartile range; SD, standard deviation; HCP, high compartment pressure; BMI, body mass index; TFA, tibia-fascia angle; ΔTFA, tibia-fascia angle difference.

Measurement reproducibility was excellent, with ICCs of 0.815 (95% CI: 0.739–0.871) for injury TFA and 0.887 (95% CI: 0.807–0.930) for ΔTFA.

### Association between ΔTFA and ICP

**Fig 3:**
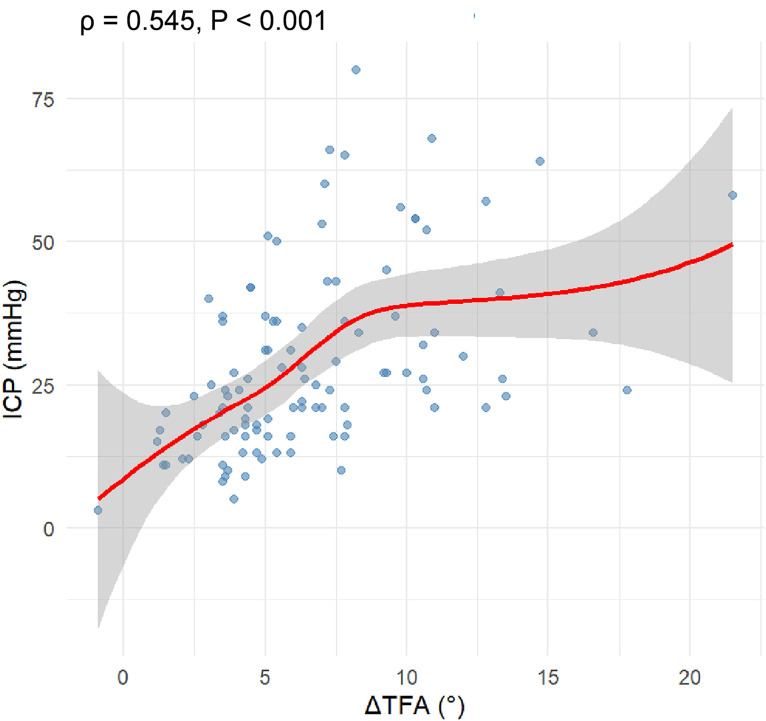
Correlation between tibia-fascia angle difference (ΔTFA) and intracompartmental pressure (ICP). The scatter plot illustrates a moderate positive correlation between the two parameters.

Spearman correlation analysis showed a moderate positive monotonic association between ΔTFA and ICP (*ρ* = 0.545, *P* < 0.001; Fig 3). Multiple linear regression analysis further revealed a significant positive association (*β* = 1.74, 95% CI: 1.06–2.43, *P* < 0.001), which remained robust after heteroscedasticity correction (Table 2). Model diagnostics indicated excellent multicollinearity control (all VIF < 1.4) with mild residual non-normality (Shapiro-Wilk *P* = 0.017) and borderline heteroscedasticity (Breusch-Pagan *P* = 0.045). Interaction analyses showed no significant effect modification by Schatzker classification or injury mechanism (all *P* > 0.100). Subgroup analyses demonstrated significant ΔTFA-ICP correlations in females (*β* = 1.82, *P*_adj_ < 0.001), males (*β* = 1.43, *P*_adj_ = 0.018), and Schatzker Ⅳ-Ⅵ fractures (*β* = 2.11, *P*_adj_ < 0.001), but not in Schatzker I-III fractures (*β* = 1.07, *P*_adj_ = 0.101).

**Table 2:** Multivariable linear regression analysis of factors associated with intracompartmental pressure.

Variable	Standard Regression		Robust Regression^a^	
	*β* (95% CI)	*P*-value^b^	*β* (95% CI)	*P*-value^b^
**ΔTFA**	1.74 (1.06, 2.43)	**<0.001**	1.74 (1.07, 2.41)	**<0.001**
**Age**	−0.32 (−0.55, −0.10)	**0.006**	−0.29 (−0.53, −0.05)	**0.018**
**Male** ^c^	−1.11 (−6.85, 4.63)	0.702	−1.42 (−6.79, 3.95)	0.601
**BMI**	−0.34 (−0.99, 0.32)	0.313	−0.30 (−0.91, 0.30)	0.326
**Schatzker Ⅳ-Ⅵ** ^c^	4.47 (−0.76, 9.70)	0.093	4.80 (−0.21, 9.82)	0.060
**Blunt trauma** ^c^	12.40 (2.36, 22.44)	**0.016**	9.85 (−3.52, 23.21)	0.147
**Electric bicycle accident** ^c^	1.29 (−5.31, 7.89)	0.700	−0.07 (−6.56, 6.41)	0.982
**Motor vehicle accident** ^c^	−1.24 (−7.62, 5.13)	0.700	−2.42 (−8.08, 3.24)	0.398

a Robust regression results using MM-estimation with HC3 standard errors.

b *P*-value<0.05 was considered for statistical significance (significant values in bold).

c Reference categories: Female (for Sex), Schatzker I–III (for Classification), Fall (for Injury mechanism).

*β*, unstandardized beta coefficients; CI, confidence interval; BMI, body mass index; ΔTFA, tibia-fascia angle difference; ICP, intracompartmental pressure.

### Diagnostic performance of ΔTFA for HCP

ROC curve analysis demonstrated moderate diagnostic accuracy of ΔTFA for detecting HCP, with an area under the curve (AUC) of 0.716 (95% CI: 0.621–0.812). At the optimal threshold of ΔTFA ≥4.9°, sensitivity reached 86.5% and specificity 52.9%. The positive and negative predictive values were 50.0% and 87.8%, respectively (Table 3).

**Table 3:** Diagnostic performance of ΔTFA for identifying high compartment pressure.

Metric	AUC (95% CI)	Optimal ΔTFA	Sensitivity	Specificity	PPV	NPV
**Value**	0.716 (0.621–0.812)	4.9°	86.5%	52.9%	50.0%	87.8%

AUC, area under the curve; ΔTFA, tibia-fascia angle difference; PPV, Positive Predictive Value; NPV, Negative Predictive Value.

## Discussion

This study represents the first clinical validation of ultrasound-derived ΔTFA as a potential noninvasive indicator of ICP in tibial plateau fractures. We demonstrated a strong positive correlation between ΔTFA and invasive ICP (*β* = 1.74, *P* < 0.001), with ΔTFA showing moderate diagnostic accuracy for HCP (AUC = 0.716). Unlike traditional invasive monitoring, this morphometric approach offers a rapid, ionizing-radiation-free assessment that leverages the uninjured limb as an internal physiological reference. While sensitivity was high (86.5%), the modest specificity (52.9%) suggests ΔTFA may serve best as a screening tool.

Our findings reveal critical distinctions from Mühlbacher et al.’s cadaveric study [25], which reported a 4 mmHg/° TFA-ICP relationship. While both studies confirm a linear association, our clinical cohort demonstrated a markedly lower slope (*β* = 1.74, *P* < 0.001). This discrepancy likely stems from inherent physiological differences. Cadaveric fascia is dehydrated and rigid, whereas living tissue retains viscoelasticity and muscle tone; In living subjects, compensatory vascular responses and muscle compliance may dampen fascial displacement per unit of pressure rise [34,35]. Furthermore, Mühlbacher’s model, using saline-infused cadavers (median age: 81 years), could not account for age-related muscle atrophy or dynamic vascular responses [36–39], whereas our inclusion of younger trauma patients (median age: 45 years) with contralateral reference limbs minimized anatomical variability. Notably, their reported interindividual TFA variation aligns with our clinical observations, reinforcing the necessity of side-to-side comparisons. These findings underscore the necessity of clinical validation over direct extrapolation from ex vivo models.

Our findings further demonstrate that ultrasound-derived ΔTFA may serve as a potential noninvasive adjunctive screening parameter for HCP. Current mainstream noninvasive techniques predominantly rely on strain elastography (SE) or shear-wave elastography (SWE). While robust ICP-SWE correlations exist in animal models, as evidenced by Zhang et al.’s rabbit study (*r* = 0.942) [1] and Ren et al.’s turkey infusion experiments (*r* = 0.802) [12], these methods require specialized hardware and face operational constraints in routine clinical practice. Critically, ΔTFA addresses a key limitation identified in prior human studies. Wang et al. [40] reported no correlation between compartment thickness and ICP in fracture patients (*P* < 0.001), whereas our technique quantifies angular fascial displacement and demonstrates significant ΔTFA-ICP correlation (*β* = 1.74, *P* < 0.001). Unlike SWE techniques, which exhibit interindividual variability in healthy cohorts [6], or SE methods necessitating external compression [16], ΔTFA leverages standardized B-mode imaging integrated with contralateral reference measurements. This strategy delivers a quantifiable parameter to address diagnostic gaps left by dimensional assessments alone while maintaining practical utility for rapid, noninvasive screening in acute trauma settings.

Clinically, the high sensitivity (86.5%) but moderate specificity (52.9%) of ΔTFA suggests its optimal role is that of a “rule-out” screening tool rather than a standalone diagnostic. An elevated ΔTFA (≥4.9°) warrants heightened vigilance or confirmatory invasive monitoring, whereas a low value may reassure clinicians in equivocal cases. However, we caution against using the 4.9° cutoff as a rigid dichotomy. Given the potential for minor angular measurement errors to alter classification near the threshold, serial monitoring of ΔTFA trends may provide greater diagnostic value than a single static measurement. This aligns with modern ACS management strategies that prioritize trend analysis over absolute pressure thresholds.

Interpretation of these results requires recognition of anatomical constraints. The valid correlation established herein is specific to the anterior compartment. Extrapolation to the deep posterior compartment, bounded by the interosseous membrane and deep transverse fascia, is scientifically invalid due to its complex, non-compliant architecture. Similarly, the dependence on a healthy contralateral reference precludes the use of this method in patients with bilateral lower extremity injuries.

Several limitations of this study warrant consideration. First, the single-center design and sample size may restrict generalizability. Second, although inter-observer reliability was excellent, the technique remains operator-dependent. Third, the lack of serial measurements precluded assessment of its dynamic monitoring value. Therefore, broader validation is required before clinical adoption. Future work should extend cadaveric and clinical studies to other compartments, validate ΔTFA in multicenter cohorts with diverse ACS etiologies, incorporate AI-assisted angle quantification to enhance reproducibility, and examine dynamic ΔTFA changes and their associations with tissue perfusion.

## Conclusions

This observational study further explored ultrasound-derived ΔTFA as a rapid, noninvasive method for ICP assessment in tibial plateau fractures. Our study demonstrated a significant linear relationship between ΔTFA and invasive ICP and validated its moderate diagnostic accuracy for HCP. These findings suggest the potential utility of ΔTFA as a screening tool for ACS in resource-limited settings, particularly in patients with high-risk Schatzker type Ⅳ-Ⅵ fractures, though it remains unvalidated for other compartments and in cases of bilateral limb injury. Future multicenter studies should refine ΔTFA thresholds and integrate AI-assisted quantification to enhance clinical adoption.
